# An analysis of Covid-19 deaths and equality in Northern Ireland.

**DOI:** 10.23889/ijpds.v7i3.1871

**Published:** 2022-08-25

**Authors:** John Hughes, Jos Ijpelaar, Rita McAuley, Ian Shuttleworth, Estelle Lowry, Deborah Lyness

**Affiliations:** 1 NISRA (ADR-NI); 2 Queen's University

## Objectives

The overarching aim is to extend understanding of Covid-19 and non Covid-19 mortality during the pandemic. For the first two waves of the pandemic in Northern Ireland, the study addressed evidence gaps for both Covid-19 and non Covid-19 deaths in relation to equality group, health and socio-demographic characteristics.

## Approach

Prior to this research, mortality analyses in Northern Ireland during the pandemic had been largely based on information recorded on death certificates and information gaps remained. This research linked death records to extensive socio-demographic, health and equality group information, retrieved from the 2011 Census, providing a more comprehensive assessment of mortality.

The research approach demonstrated innovative use of linked datasets in support of public policy. Findings also complement similar analysis published by the Office for National Statistics (ONS). Research questions were shaped and informed by information queries from a range of stakeholders including the Department of Health and elected officials.

## Results

Results published to date are based on Covid-19 and non Covid-19 deaths occurring in the 7-month period between 1st March 2020 and 30th September 2020. We found that there was a 48% and 40% higher risk for persons self-reporting having a disability at the time of the 2011 Census (compared to ‘non-disabled’ people) for Covid-19 and non Covid-19 mortality respectively. After accounting for differences in age, sex and area of residence, there was no significant difference in risk of Covid-19 death, for the time period March to September 2020, for those who identified as Catholic at the time of the 2011 Census, compared to Protestants and other Christians.

## Conclusion and Relevance

The research addresses priority evidence gaps identified by government officials and third sector policy leads. The depth of commentary provided within the report is enabling researchers and policy makers to gain greater familiarity with a key research resource. This will be instrumental in shaping future research through ADR Northern Ireland.

